# Acoustic and Facial Features From Clinical Interviews for Machine Learning–Based Psychiatric Diagnosis: Algorithm Development

**DOI:** 10.2196/24699

**Published:** 2022-01-24

**Authors:** Michael L Birnbaum, Avner Abrami, Stephen Heisig, Asra Ali, Elizabeth Arenare, Carla Agurto, Nathaniel Lu, John M Kane, Guillermo Cecchi

**Affiliations:** 1 Department of Psychiatry The Zucker Hillside Hospital Northwell Health Glen Oaks, NY United States; 2 The Feinstein Institute for Medical Research Northwell Health Manhasset, NY United States; 3 The Donald and Barbara Zucker School of Medicine at Hofstra/Northwell Hempstead, NY United States; 4 Computational Biology Center IBM Research Yorktown Heights, NY United States; 5 Icahn School of Medicine at Mount Sinai New York City, NY United States

**Keywords:** audiovisual patterns, speech analysis, facial analysis, psychiatry, schizophrenia spectrum disorders, bipolar disorder, symptom prediction, diagnostic prediction, machine learning, audiovisual, speech, schizophrenia, spectrum disorders

## Abstract

**Background:**

In contrast to all other areas of medicine, psychiatry is still nearly entirely reliant on subjective assessments such as patient self-report and clinical observation. The lack of objective information on which to base clinical decisions can contribute to reduced quality of care. Behavioral health clinicians need objective and reliable patient data to support effective targeted interventions.

**Objective:**

We aimed to investigate whether reliable inferences—psychiatric signs, symptoms, and diagnoses—can be extracted from audiovisual patterns in recorded evaluation interviews of participants with schizophrenia spectrum disorders and bipolar disorder.

**Methods:**

We obtained audiovisual data from 89 participants (mean age 25.3 years; male: 48/89, 53.9%; female: 41/89, 46.1%): individuals with schizophrenia spectrum disorders (n=41), individuals with bipolar disorder (n=21), and healthy volunteers (n=27). We developed machine learning models based on acoustic and facial movement features extracted from participant interviews to predict diagnoses and detect clinician-coded neuropsychiatric symptoms, and we assessed model performance using area under the receiver operating characteristic curve (AUROC) in 5-fold cross-validation.

**Results:**

The model successfully differentiated between schizophrenia spectrum disorders and bipolar disorder (AUROC 0.73) when aggregating face and voice features. Facial action units including cheek-raising muscle (AUROC 0.64) and chin-raising muscle (AUROC 0.74) provided the strongest signal for men. Vocal features, such as energy in the frequency band 1 to 4 kHz (AUROC 0.80) and spectral harmonicity (AUROC 0.78), provided the strongest signal for women. Lip corner–pulling muscle signal discriminated between diagnoses for both men (AUROC 0.61) and women (AUROC 0.62). Several psychiatric signs and symptoms were successfully inferred: blunted affect (AUROC 0.81), avolition (AUROC 0.72), lack of vocal inflection (AUROC 0.71), asociality (AUROC 0.63), and worthlessness (AUROC 0.61).

**Conclusions:**

This study represents advancement in efforts to capitalize on digital data to improve diagnostic assessment and supports the development of a new generation of innovative clinical tools by employing acoustic and facial data analysis.

## Introduction

Approximately 20% of individuals aged 15 years and older experience psychiatric illness annually [1-3]. Psychiatrists may see as many as 8 patients hourly and are often unable to obtain the detailed information necessary to make effective, evidence-based, and personalized clinical decisions [4-6]. In contrast to all other areas of medicine, psychiatry is still nearly entirely reliant on subjective assessments such as patient self-report and clinical observation [7,8]. There are few valid and reliable tests, biomarkers, and objective sources of collateral information available to support diagnostic procedures and assess health status. The lack of objective information on which to base clinical decisions can contribute to reduced quality of care, underrecognized signs and symptoms, and poorer treatment outcomes, including higher dropout rates, reduced medication adherence, and persistent substance abuse [9,10]. Behavioral health clinicians need access to objective and reliable, easily collected, and interpretable patient data to enable quick, effective, and targeted interventions [11,12].

In recent years, progress has been made in audiovisual data processing [13-21]. Advances in this technology could play a pivotal role in supporting automated methods of collecting objective adjunctive patient data to inform diagnostic procedures, psychiatric symptom identification, and psychiatric symptom monitoring. Speech analysis, in particular, has been studied [22-36] because changes in both the content and acoustic properties of speech are known to be associated with several psychiatric conditions: disorganized speech in schizophrenia, pressured speech in mania, and slowed speech in depression [7]. Moreover, speech represents a universal, easily extracted, and clinically meaningful biological process and is therefore well positioned to serve as an objective marker of psychiatric illness [27]. Prior research has demonstrated the potential for the use of speech properties to distinguish between individuals with and without a variety of psychiatric disorders with high degrees of accuracy [22-36]. Acoustic analysis, for instance, has demonstrated that participants with schizophrenia tend to exhibit less total time talking, reduced speech rate, and higher pause duration [23,27,33-40] than healthy participants and that participants with bipolar disorder demonstrate increases in tonality [41-43].

Concurrently, alterations in facial expressivity accompany several psychiatric illnesses: flat or inappropriate affect in individuals with schizophrenia, euphoric or labile affect in mania, and slowed or diminished facial movements in depression [7]. Video analysis has accordingly emerged as a potentially objective and reliable method for capturing subtle head, face, and eye movements with greater precision than by clinical observation alone [16,44-46]. Alterations in facial expressivity have demonstrated success in predicting the presence of various psychiatric illnesses including schizophrenia spectrum disorders [47-49], mood disorders [49-51], and autism spectrum disorders [48].

Audiovisual patterns represent an easily extractable, naturalistic, universal, and objective data that could serve as viable digital biomarkers in psychiatry, contributing adjunctive information about a patient, beyond what can be assessed solely through traditional means. No study, to the best of our knowledge, has explored the potential for using audiovisual data to discriminate between a diagnosis of schizophrenia or bipolar disorder, a task which can be challenging for behavioral health clinicians given significant symptom overlap [52,53], especially during the early course of illness development. Additionally, few studies [19,54] have explored the relationship between audiovisual data and psychiatric symptoms, commonly used as primary outcome measures, to more efficiently and more effectively identify the presence of a specific psychiatric sign or symptom. Furthermore, research thus far has largely explored individual data sources in isolation [19,20], however, advancing this critical work will now require integrating multiple streams of digital data.

We aimed to differentiate between schizophrenia spectrum disorders and bipolar disorder using audiovisual data alone. We hypothesized that physiological data from voice acoustics and facial action units could be used to distinguish between individuals with schizophrenia spectrum disorders and individuals with bipolar disorder and that these signals would be associated with specific psychiatric signs and symptoms.

## Methods

### Recruitment

Participants between the ages of 15 and 35 years old diagnosed with schizophrenia spectrum disorders or bipolar disorder were recruited from Northwell Health Zucker Hillside Hospital’s inpatient and outpatient psychiatric departments. Diagnoses were based on clinical assessment of the most recent episode and were extracted from participant’s medical record at the time of consent. Most participants with schizophrenia spectrum disorders were recruited from the Early Treatment Program, which is a specialized outpatient early psychosis intervention clinic. Individuals with psychiatric comorbidities (such as substance use disorders) were included. Participants with known physical impairments (such as paralysis or severe laryngitis) capable of impacting facial movements or acoustic capabilities were excluded. Eligible participants were recruited by a research staff member. Healthy volunteers who had already been screened for prior studies were also recruited. Recruitment occurred between September 2018 and July 2019. The study was approved by the institutional review board (18-0137) of Northwell Health. Written informed consent was obtained from adult participants and legal guardians of participants under 18 years. Assent was obtained from minors. All participants received treatment as usual.

### Interviews

Participants were assessed at baseline and invited to return for optional quarterly assessments thereafter for a maximum of 12 months. Healthy volunteers were assessed at baseline and invited to return for optional assessments at month 6 and month 12. At each visit, all participants, including healthy volunteers, were interviewed by a trained and reliable research rater utilizing the Brief Psychiatric Rating Scale (BPRS) [55], Scale for the Assessment of Negative Symptoms (SANS) [56], Hamilton Depression Rating Scale (HAMD) [57], and Young Mania Rating Scale (YMRS) [58]. In addition, at each visit, participants were asked a series of 5 emotionally neutral, open-ended questions designed to encourage speech production. For example, participants were asked to describe a typical dinner, discuss a television show or movie that they had watched, or talk about a current or prior pet. Participants were instructed to talk freely and prompted to continue to talk as much as they liked for each response. Similar methods for speech extraction have been successfully implemented in prior research [34]. Both participant and the interviewer wore headsets with microphones connected to a 2 by 2 amplifier (TASCAM) to record audio. Video was recorded with an iPad Pro (Apple Inc) focused on participants’ facial expressions.

Raw data were stored in a firewalled server and were never shared outside of Northwell Health. The processing of high-level features was implemented locally, and only those features were used for further analysis outside the raw data server. High-level feature data remained within Health Insurance Portability and Accountability Act–compliant servers.

### Data Preprocessing

Before extracting acoustic features, saturation, if present, was removed by identifying time points with amplitudes higher than 99.99% of the maximum value, and given that recordings involved the use of two audio channels (one each, for participant and interviewer), we extracted only the participant’s voice.

Acoustic features were extracted using the OpenSMILE open-source toolbox [59]. We used a predefined feature set [60] for low-level descriptors. This configuration encompasses 150 features, which were computed with a fixed window size (ie, mel-frequency cepstral coefficients -25 ms) but with a sampling rate of 10 ms (Multimedia Appendix 1).

For facial features, we used openFace software [61]. This tool detects the presence and intensity of 18 facial expressions called action units (Multimedia Appendix 2). The video sampling rate was 30 Hz.

Both facial action units and acoustic time series were downsampled to 10 Hz (by taking the average value in each consecutive 0.1-second window) and aligned. We then fragmented each interview into consecutive 1.5-minute blocks. In each block, we derived 2 sets of aggregate features (one that was computed when the participant was listening, the other while speaking) to help ensure that the silence between answers did not have an effect on acoustic feature values and that the dynamics of facial action units in both conditions were captured by the models. Mean value and standard deviation were computed for each feature and for each 1.5-minute block. For better classification generalization and to reduce overfitting, we augmented each interview 25 times by selecting only 1 out of 2 consecutive blocks randomly for each block in the sequence.

### Classification Tasks

We explored 2 main classification tasks: differential diagnosis, assigning an interview as belonging to a specific group (either schizophrenia spectrum disorders or bipolar disorder) based purely on physiological patterns, and symptom detection, predicting the presence of a psychiatric sign or symptom. In total, 75 classification tasks were run, each corresponding to the 75 unique psychiatric signs and symptoms assessed with the BPRS (18 items), SANS (22 items), YMRS (11 items), and HAMD (24 items). For each classification task, participants were assigned to the positive class if their symptom score exceeded the clinical threshold of at least mild severity: score ≥3 on BPRS items (range 1-7), score ≥2 on SANS items (range 0-5), score ≥2 or ≥4 on YMRS items (with ranges 0-4 and 0-8, respectively), and score ≥2 or ≥1 on HAMD items (with ranges 0-4 and 0-2, respectively). Total scores could range from 18 to 126 for the BPRS, 0 to 110 for the SANS, 0 to 60 for the YMRS, and 0 to 76 for the HAMD.

For each classification task, we computed 2 independent models for both men and women. This was done to prevent possible sex-specific physiological confounds in voice and face to impact the results, as the bipolar disorder group was composed of a majority of women. Additionally, we aimed to build models that were not individual-dependent.

All inferences were undertaken using a gradient boosting classifier [62] (Python; Scikit-learn library [63]) (fixed seed 0, deviance loss, 0.1 learning rate, 100 weak learners, with 10% of all samples selected randomly used for fitting the individual base learners). All inferences were run in stratified 5-fold cross-validation (participants were divided in 5 nonoverlapping groups and each group was used once as a validation, while the 4 remaining groups formed the training set). Only the most predictive features—those achieving a leave-one-out area under the receiver operating characteristic curve [AUROC] greater than 0.6 on the training set of each fold—were used by the gradient boosting classifier.

Finally, we ensured that each group (both in the positive and negative class) had similar average interview durations, We removed the final few minutes from the end of the lengthier interviews (corresponding to the difference between the average length in each class) to ensure that interview duration was not a confounding factor in classification performance, because longer interviews would provide greater statistical sampling of the features.

### Aggregating Different Modalities

We investigated 3 different models including a Face model (all relevant facial action units features), a Voice model (all relevant acoustic features), and a Face–Voice model, which was constructed by averaging the probability outputs of the Face model and the Voice model. For each inference, 5-fold AUROC, accuracy, accuracy chance (the accuracy one would get by randomly attributing the classes), and F scores (for both classes of the classification) were calculated. A threshold of 0.5 was used to compute accuracy and F scores. To rank features (to assess which ones were most predictive), we used a 5-fold AUROC for each feature sequence alone. We report the most successful models per modality (voice alone, face alone, or combined voice and face).

## Results

### General

In total, 89 participants (mean age 25.3 years; male: 48/89, 53.9%; female: 41/89, 46.1%) with schizophrenia spectrum disorders (n=41), bipolar disorder (n=21), and healthy volunteers (n=27) were included (Table 1), resulting in 146 interviews (mean 1.64, SD 0.84 interviews per participant). Total scores (representing aggregate scores from individual items) indicated that participants were predominantly in remission at the time of the assessments (Table 2); however, several participants scored moderate or severe on 1 or more items in the BPRS (schizophrenia spectrum disorders: 22/41, 54%; bipolar disorder: 8/21, 38%), SANS (schizophrenia spectrum disorders: 33/41, 80%; bipolar disorder: 14/21, 67%), YMRS (schizophrenia spectrum disorders: 18/41, 44%; bipolar disorder: 8/21, 38%), and HAMD (schizophrenia spectrum disorders: 32/41, 78%; bipolar disorder: 10/21, 48%). Participant assessments, including speech extraction and symptom rating scales, lasted a mean duration of 27 minutes (SD 11).

**Table 1:** Demographic and clinical characteristics.

Characteristic			Schizophrenia spectrum disorders (n=41)		Bipolar disorder (n=21)		Healthy volunteers (n=27)		Full sample (n=89)
Age (in years), mean (SD)			23.7 (3.97)		25.3 (4.24)		28.5 (5.15)		25.5 (4.83)
**Sex, n (%)**									
	Male	29 (71)		7 (33)		12 (44)		48 (54)	
	Female	12 (29)		14 (67)		15 (56)		41 (46)	
**Race/ethnicity, n (%)**									
	African American/Black	24 (58)		3 (14)		8 (30)		35 (39)	
	Asian	6 (15)		4 (19)		6 (22)		16 (18)	
	Caucasian	10 (24.)		9 (43)		10 (37)		29 (33)	
	Mixed race/other	1 (2)		5 (24)		2 (7)		8 (9)	
	Pacific Islander	0 (0)		0 (0)		1 (4)		1 (1)	
	Hispanic	5 (12)		3 (14)		1 (4)		9 (10)	
**Diagnosis (most recent episode), n (%)**									
	Schizophrenia	19 (46)		N/A^a^		N/A		19 (21.)	
	Schizophreniform	10 (24)		N/A		N/A		10 (11)	
	Schizoaffective	7 (17)		N/A		N/A		7 (8)	
	Unspecified schizophrenia spectrum disorders	5 (12)		N/A		N/A		5 (6)	
	Bipolar disorder (manic)	N/A		16 (76)		N/A		16 (18)	
	Bipolar disorder (depressed)	N/A		3 (14)		N/A		3 (3)	
	Bipolar disorder (mixed)	N/A		2 (10)		N/A		2 (2)	
**Interviews, n**									
	Baseline	41		21		27		89	
	Follow up	33		17		7		57	
Interview length, mean (SD)			29.5 (13.1)		29.5 (9.3)		20.7 (6.1)		27 (11)

^a^N/A: not applicable.

**Table 2:** Symptom rating scale scores for diagnostic and sex groups.

Group			Brief Psychiatric Rating Scale score^a^, mean (SD)		Scale for the Assessment of Negative Symptoms score^b^, mean (SD)		Young Mania Rating Scale score^c^, mean (SD)		Hamilton Depression Rating Scale score^d^, mean (SD)
**Schizophrenia spectrum disorders**									
	All	26.5 (6.8)		22.6 (12.3)		3.9 (3.6)		8.7 (6.3)	
	Men	28.1 (7.0)		25.5 (11.2)		4.6 (3.8)		9.8 (6.7)	
	Women	22.8 (4.4)		15.8 (12.1)		2.3 (2.1)		6.0 (4.1)	
**Bipolar disorder**									
	All	26.8 (8.3)		14.0 (9.2)		7.5 (8.5)		9.4 (7.9)	
	Men	25.9 (5.7)		10.5 (8.8)		8.9 (9.1)		9.8 (10.3)	
	Women	27.3 (9.5)		16.2 (8.7)		6.7 (8.1)		9.2 (5.9)	

^a^The total score can range from 18-126.

^b^The total score can range from 0-110.

^c^The total score can range from 0-60.

^d^The total score can range from 0-76.

### Differential Diagnosis

**Figure 1:**
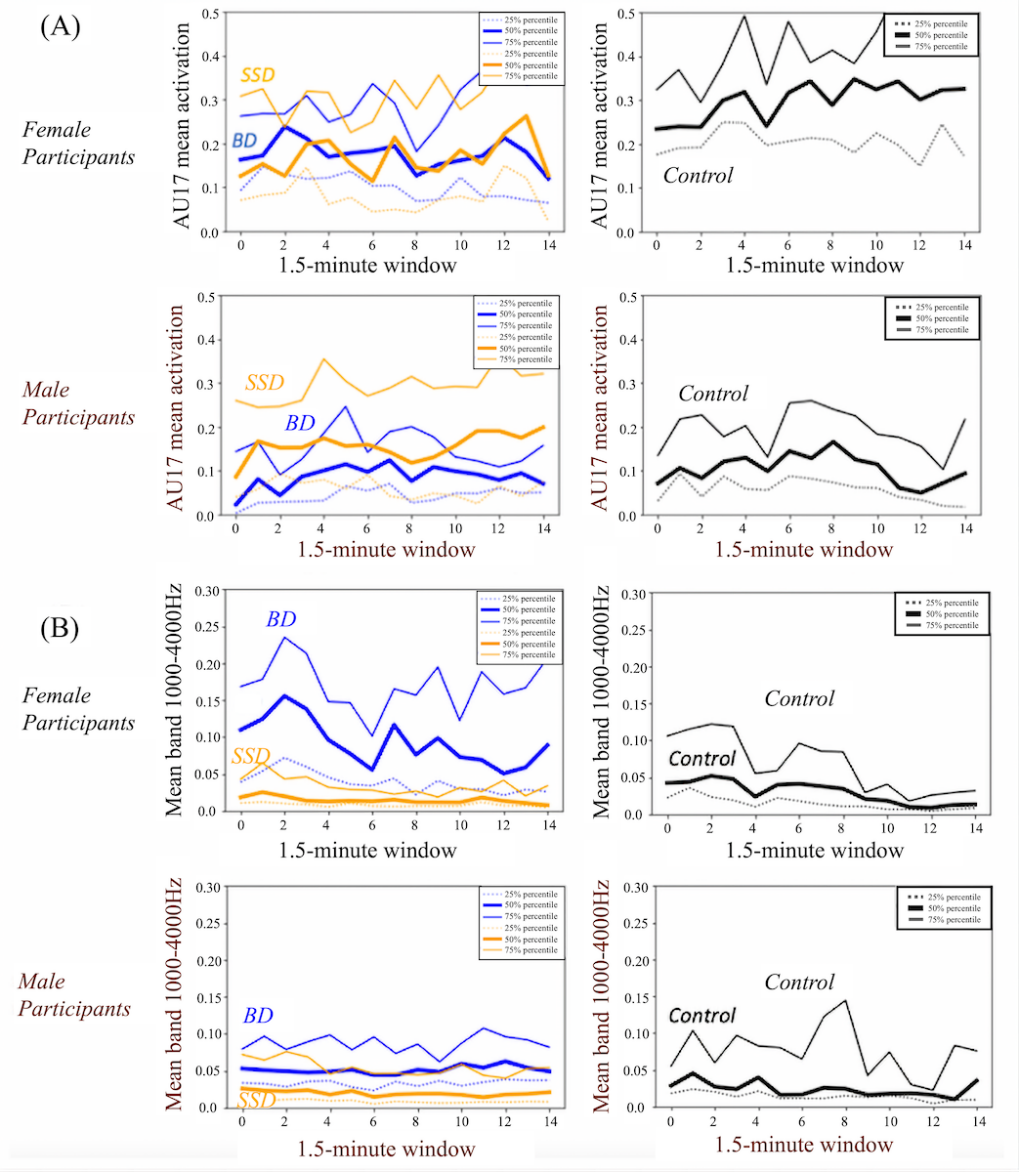
Sex-specific features that discriminate between schizophrenia spectrum disorders and bipolar disorder: (A) mean activation of AU17 (chin raising while speaking), and (B) mean value of the energy in the frequency band 1-4 kHz. BD: bipolar disorder; SSD: schizophrenia spectrum disorders.

Differential diagnosis classification performed well (5-fold AUROC 0.73) when aggregating features from both face and voice (Table 3). Facial action units, such as AU17 (Figure 1A), provided the strongest signal in discrimination between men with schizophrenia spectrum disorders and men with bipolar disorder. Men with schizophrenia spectrum disorders activated their chin-raising muscle (AU17: 5-fold AUROC 0.74) and lip corner–pulling muscle (AU12: 5-fold AUROC 0.61) more frequently than men with bipolar disorder, while demonstrating reduced activation of their cheek-raising muscle (AU6: 5-fold AUROC 0.64). In contrast, voice features, such as mean energy in the in the frequency band 1-4 kHz (Figure 1B), performed best for women. Women with schizophrenia spectrum disorders demonstrated reduced energy in the frequency band 1-4 kHz (5-fold AUROC 0.80), reduced spectral harmonicity (5-fold AUROC 0.78), and increased spectral slope (5-fold AUROC 0.77) compared with women with bipolar disorder. When comparing participants with schizophrenia spectrum disorders to healthy volunteers and bipolar disorder to healthy volunteers, we achieved a 5-fold AUROC of 0.78 for both classification tasks.

**Figure 2:**
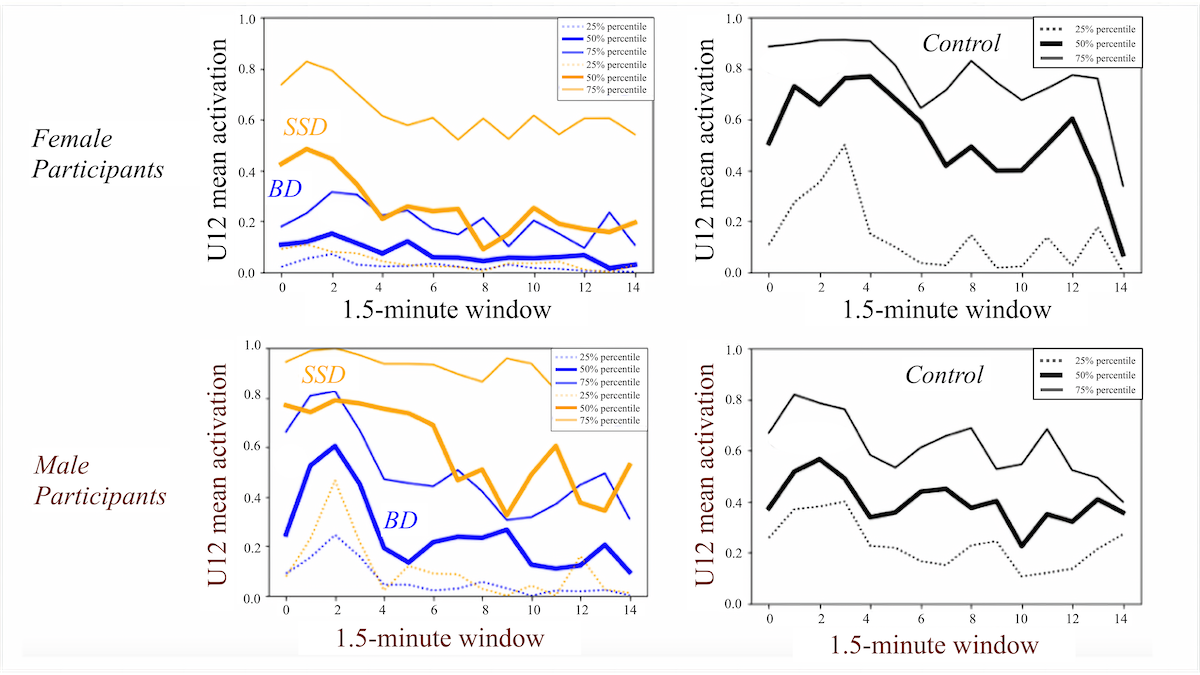
AU12 (lip-corner pulling while speaking) feature. For each signal, the 25th percentile, median, and 75th percentile values are shown for each 1.5-minute window. Bipolar disorder is represented in blue, schizophrenia spectrum disorders is represented in yellow, and on the adjacent plot, healthy volunteers is represented in black. BD: bipolar disorder; SSD: schizophrenia spectrum disorders.

We identified some features that discriminated well between schizophrenia spectrum disorders and bipolar disorder across both sexes: lip-corner pulling (AU12), which represented the movement of lip corners pulled diagonally by the zygomaticus major muscle (5-fold AUROC men: 0.61; women: 0.62) for which the mean value was higher on average for participants with schizophrenia spectrum disorders than for participants with bipolar disorder (Figure 2). The timing of this feature was observed to be important to classification performance—AU12 values were higher on average at the beginning of the interview and decreased over time.

**Table 3:** Diagnostic classification.

Features	AUROC^a^	Accuracy	Accuracy chance	F score	
				Schizophrenia spectrum disorders	Bipolar disorder
Voice	0.65	0.71	0.55	0.80	0.46
Face	0.68	0.72	N/A^b^	0.80	0.56
Face and voice	0.73	0.72	N/A	0.80	0.56

^a^AUROC: area under the receiver operating characteristic curve.

^b^N/A: not applicable.

### Symptom Classification

Best performing models were derived from the SANS scale, predominantly from the affective flattening and blunting subgroup (global affective flattening, vocal inflection, paucity of expression, unchanging facial), avolition/apathy subgroup (physical anergia, role function level, global avolition), and asociality/anhedonia subgroup (sexual interest, asociality, intimacy). Two items passed the performance threshold from the BPRS (blunted affect and motor retardation), and 2 others were derived from the HAMD scale (work interest and worthlessness). No signs or symptoms from the YMRS passed the performance threshold criteria.

Voice outperformed facial action units for blunted affect (5-fold AUROC 0.81), whereas facial action units outperformed voice for unchanging facial expression (5-fold AUROC 0.64) (Table 4). Synergy between both modalities was observed for paucity of expression (5-fold AUROC 0.81).

Voice alone outperformed facial action units for several items including asociality (5-fold AUROC 0.63) and work and interests (5-fold AUROC 0.64) (Table 5). Facial action units alone outperformed voice for worthlessness (5-fold AUROC 0.61). Synergy between both modalities was observed for several other symptoms including avolition (5-fold AUROC 0.72) and anergia (5-fold AUROC 0.68). Importantly, given that these symptoms represent self-reported experiences, their relationship with measured physiological signals is likely indirect and one hypothesis is that they are linked to observable symptoms. For example, we found a correlation (*r*=0.35; *P*<.001) between work and interests and blunted affect, and a correlation (*r*=0.31; *P*<.001) between avolition and affective flattening.

**Figure 3:**
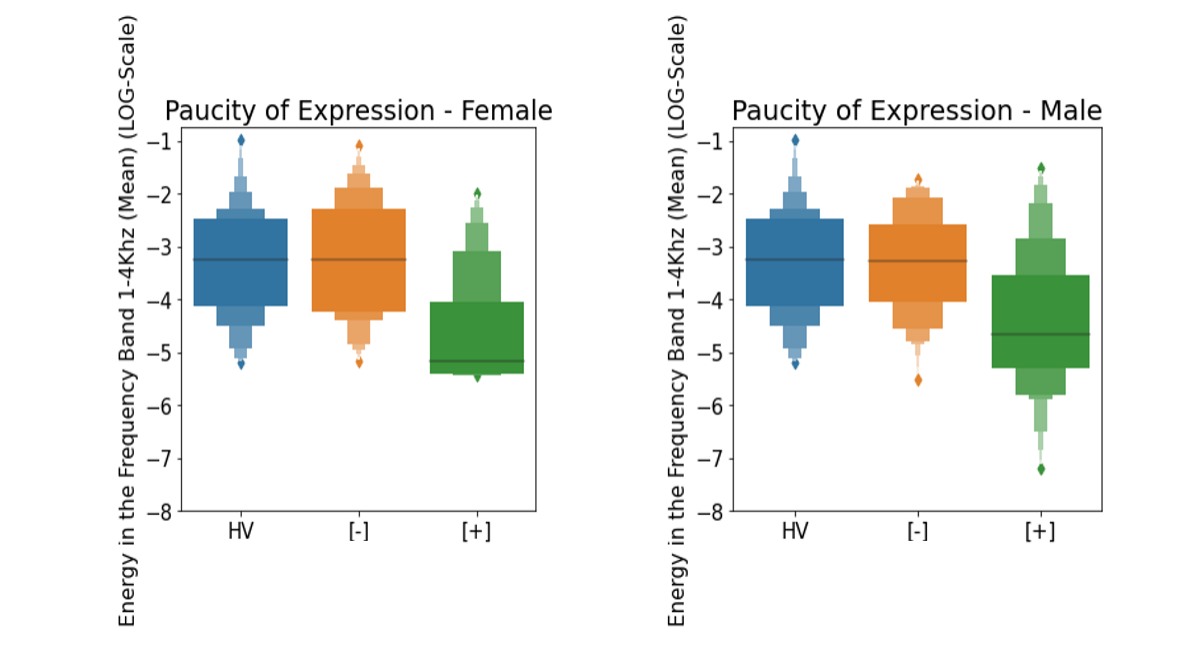
Paucity of expression score as a function of the mean value of the energy in the high frequency band 1-4 KHz (log-scale) for healthy volunteers (blue), patient participants with symptom rating scale scores below symptom threshold (orange), and patient participants with symptom rating scale scores above symptom threshold (green). A lower value of this feature is indicative of a more severe symptom across sex. The black line indicates the median value of the feature.

Among the top acoustic features (Figure 3) for objectively observed symptoms (Table 4), the mean value of the energy in the frequency band 1-4 kHz was most indicative of paucity of expression (*r*= –0.27, *P*=.004). Specifically, a reduction in the average amount of energy in high frequencies was associated with the presence of this symptom. In addition to affecting voice quality or timber (in the form vocal overtones), high frequencies (1-4 kHz) are typical in shaping consonants through rapid air motion from the mouth and through the teeth. In contrast, vowels are generally in the lower frequencies (500 Hz) and contain the majority of the voice energy. Clinically, mismatch between the acoustic frequencies of vowels and consonants jeopardizes the natural sound of the voice and leads to a reduction in speech intelligibility. This observation is stable across sex.

**Figure 4:**
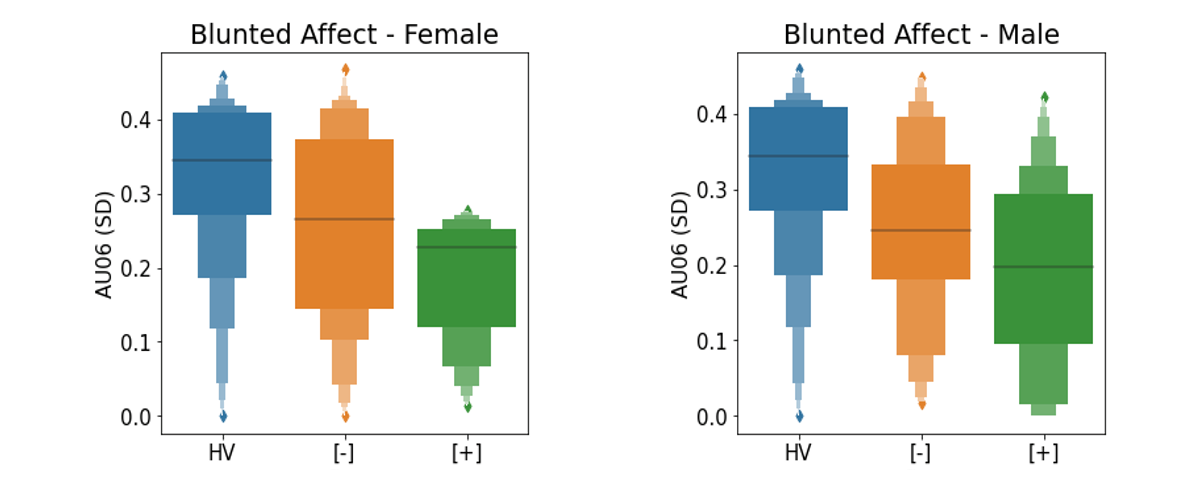
Blunted affect score as a function of the standard deviation of cheek raising (AU06) for healthy volunteers (blue), patient participants with symptom rating scale scores below symptom threshold (orange), and patient participants with symptom rating scale scores above symptom threshold (green). A lower value of this feature is indicative of a more severe symptom across sex. The black line indicates the median value of the feature.

Among the top facial action unit features (Figure 4) for the objectively observed symptoms, the standard deviation of cheek raising muscle activation, often activated to form a smile, was most indicative of blunted affect for both men and women (*r*= –0.26, *P*=.002 during speaking). When the symptom is present, the standard deviation of this feature is decreased.

**Figure 5:**
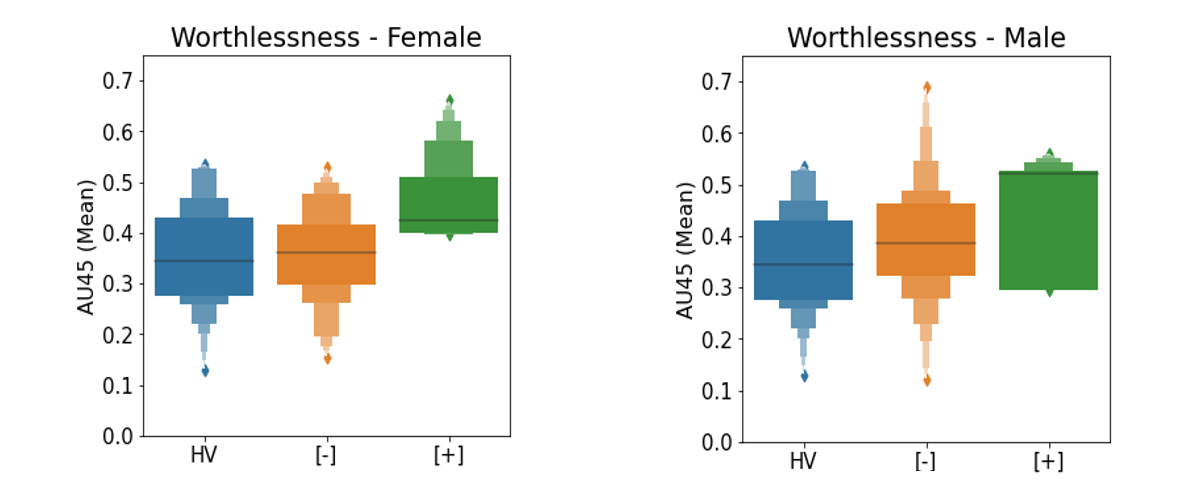
Worthlessness score as a function of the mean value of blinking (AU45) for healthy volunteers (blue), patient participants with symptom rating scale scores below symptom threshold (orange), and patient participants with symptom rating scale scores above symptom threshold (green). A higher value of this feature is indicative of a more severe symptom across sex. The black line indicates the median value of the feature.

Among the top features for self-reported symptoms (Table 5), the mean value of AU45 (blinking) during speaking is higher when the symptom feature worthlessness is present (*r*=0.30, *P*=.001, calculated over all participants) (Figure 5).

**Table 4:** Objectively observed item classification.

Symptom			Modality		AUROC^a^		Accuracy (random)		F score		
									Above clinical threshold		Below clinical threshold
**Brief Psychiatric Rating Scale**											
	Blunted affect	Voice		0.81		0.95 (0.87)		0.40 |		0.97	
	Motor retardation	Face		0.68		0.94 (0.88)		0.36		0.97	
**Scale for the Assessment of Negative Symptoms**											
	Paucity of expression	Voice, face		0.81		0.80 (0.66)		0.42		0.88	
	Global affective flattening	Voice, face		0.79		0.82 (0.71)		0.44		0.89	
	Lack of vocal inflection	Voice, face		0.71		0.88 (0.78)		0.43		0.94	
	Unchanging facial	Face		0.64		0.83 (0.70)		0.39		0.90	

^a^AUROC: area under the receiver operating characteristic curve.

**Table 5:** Self-reported items classification.

Symptom		Modality	AUROC^a^	Accuracy (random)	F score	
					Above clinical threshold	Below clinical threshold
**Scale for the Assessment of Negative Symptoms**						
	Global avolition	Voice, face	0.72	0.66 (0.53)	0.75	0.49
	Physical anergia	Voice, face	0.68	0.63 (0.51)	0.70	0.53
	Role function level	Voice, face	0.65	0.63 (0.58)	0.75	0.31
	Sexual interest	Voice, face	0.64	0.62 (0.52)	0.46	0.70
	Intimacy	Voice	0.64	0.63 (0.51)	0.56	0.67
	Asociality	Voice	0.63	0.60 (0.51)	0.54	0.65
**Hamilton Depression Rating Scale**						
	Work and interests	Voice	0.62	0.65 (0.52)	0.73	0.52
	Worthlessness	Face	0.61	0.88 (0.82)	0.32	0.94

^a^AUROC: area under the receiver operating characteristic curve.

## Discussion

We aimed to explore the feasibility of utilizing audiovisual data extracted from participant interviews for psychiatric diagnoses and to predict the presence of psychiatric signs and symptoms. Our results indicate that computational algorithms developed from vocal acoustics and facial action units can successfully differentiate between participants with either schizophrenia spectrum disorders or bipolar disorder, as well as identify the presence of several psychiatric signs and symptoms with high degrees of accuracy. Both acoustic and facial action unit features could be independently used to differentiate between participants with schizophrenia spectrum disorders and bipolar disorder in our data set, and integrating the two modalities produced the strongest signal, as previously seen in studies of depression [64-66], suggesting a synergistic interaction. Importantly, different top features were identified for men and women. Specifically, the strongest signals separating men with schizophrenia spectrum disorders from men with bipolar disorder were derived from facial features, while the strongest signals for women were derived from acoustic features. These physiological differences may be partially explained by different distributions of psychiatric signs and symptoms among the diagnostic categories. For example, men with schizophrenia spectrum disorders rated higher on average on the BPRS and SANS than men with bipolar disorder, while women with schizophrenia spectrum disorders on average scored lower than women with bipolar disorder on all rating scales. Alternatively, notable sex-specific variations in the prevalence, onset, symptom profiles, and outcome have been identified in the literature and have been attributed to differences in premorbid functioning, psychosocial response to symptoms, and differing levels of circulating hormones and receptors [67-70]. Audiovisual data may therefore detect subtle physiological differences unique to each sex and present in the expression of psychiatric disorders. In either scenario, sex differences are clearly of utmost importance when performing voice and facial analyses and must be taken into consideration when conducting future research.

We also identified audiovisual features common to both sexes that successfully differentiated between diagnostic categories. In line with prior work demonstrating altered facial expressivity in individuals with psychiatric disorders [47-51,54,71,72], we found that participants with schizophrenia spectrum disorders were much more likely to activate the facial muscle responsible for pulling the corners of their lips than participants with bipolar disorder. While this muscle is activated for several reasons, including the formation of certain words while speaking, it is also commonly used to form a smile. Interestingly, many patients with schizophrenia spectrum disorders, including the participants in our sample, experienced facial blunting and diminished facial expressivity, and one would, therefore, expect reduced facial activity compared to that of participants with bipolar disorder. While this finding may initially appear counterintuitive, it is important to note that the presence of blunted affect was associated with reduced variation in the cheek-raising muscle, which is also activated during the formation of a smile. Participants with schizophrenia spectrum disorders, therefore, activate lip corner–pulling muscles more than participants with bipolar disorder (perhaps to form a smile), though the range of activation of cheek movement was reduced if blunting was present. These findings warrant additional research particularly to understand the clinical significance of increased activation of certain facial muscles alongside decreased variability throughout the interview and its relationship to a diagnosis of schizophrenia spectrum disorders.

Some top features contributing to the diagnostic classification remained stable throughout the course of the interview, while others changed depending on the temporal pattern. For example, AU12 (lip-corner pulling), demonstrated a consistent downward trend for all participants, whereas the energy of the voice signal in the frequency band 1-4 kHz remained mostly flat. These same trends were noted in healthy volunteers as well, suggesting that the identified differences in facial activity and voice represent subtle pathological variations in the frequency or intensity of otherwise healthy activity. The amount of high frequency energy in the voice, for example, may represent a subtle state marker of psychiatric illness or perhaps a physiological response to certain medications, impacting speech intelligibility. Additionally, activating lip corner–pulling muscles more at the start of an assessment (perhaps to produce a smile) may represent a healthy behavior (as it was seen in the healthy volunteers population as well), though the frequency and degree of activation is what separates those with schizophrenia spectrum disorders from those with bipolar disorder.

Our findings suggest that a tool capable of extracting and analyzing audiovisual data from newly identified psychiatric patients might offer valuable collateral clinical information, supporting a more reliable approach to differential diagnoses. Accurately diagnosing someone as having either schizophrenia spectrum disorders or bipolar disorder is a critical first step in selecting appropriate medications and therapeutic interventions, and a task that is often challenging to behavioral health clinicians given significant symptom overlap [52,53], especially during the early course of illness development. Leveraging audiovisual signals holds promise to overcome many of the challenges associated with current assessment methods [73-76], including inaccuracies and biases in self-report and recall, as well as substantial time constraints that limit the ability to effectively obtain necessary clinical information. Diagnoses, however, are complex entities, based on multiple psychiatric symptoms, each likely corresponding to several unique audiovisual features that will need to be integrated to achieve an accurate and reliable measure. Furthermore, each symptom may correspond to various alterations in audiovisual characteristics depending on multiple factors including the frequency and intensity of the experience, as well as the individual experiencing them. Future research will therefore require large clinical and computerized collaborative efforts to characterize psychiatric symptoms and diagnoses in an accurate and objective manner.

Several psychiatric signs and symptom inferences were accurately made using features extracted from voice and face either individually or combined. Similar to the findings of prior studies [36,45,71], the most successful models were derived from the SANS, and greater accuracy was achieved with externally observable psychiatric signs and symptoms such as blunted affect and lack of vocal inflection. Integrating audiovisual data into symptom assessment might, therefore, offer more efficient and objective methods to identify and track changes in negative symptoms, beyond what can be achieved through traditional clinical observation alone. A more challenging task will be to provide greater objectivity to the assessment of symptoms such as hallucinations, delusions, and suicidal thoughts. In contrast to the findings of prior research, we did not find association between brow movements and delusions or depression [54,72]. One possibility is that the prevalence of negative symptoms (such as blunted affect and affective flattening) in our sample masked the expression (and, therefore, detection) of subtle physiological signals associated with these symptoms. Our findings do, however, suggest that audiovisual data can be representative of subjectively experienced symptoms, including worthlessness and avolition, though further research is required to uncover their complex correlational structure. For instance, the observed associations between audiovisual features and psychiatric symptoms may be justly considered as purely epiphenomenal, yet a mechanistic understanding of how the symptom is expressed in the feature is not obvious and may provide insights into the diagnostic conditions. When the severity of one symptom changes, it may affect the distribution of the other symptoms in a deterministic way. Consequently, it is possible to find correlations between symptoms and physiological data even if they are not causally linked. Those correlations, if confirmed in larger studies, would be very valuable as they offer indirect proxies to more subjective experiences that are not directly quantifiable. Further research is required to determine the clinical significance of physiological changes in voice and face, as well as how they might correspond to a particular psychiatric symptom to effectively incorporate audiovisual data into clinical care. A critical, though challenging, task for future research would be maximize the level of isolated psychiatric symptoms while containing other symptoms to avoid confounding the signals that we aim to capture. Accordingly, comparing participants to themselves longitudinally as symptoms fluctuate over the course of various pathological states would also help reduce potential confounds in the signals. Future research should consider how physiological differences in facial expression and voice may manifest in other clinical settings and structured tasks as well, such as emotion elicitation [77]. Lastly, follow-up studies should consider exploring participant response times, and other measures of interviewer–interviewee interaction by recording and analyzing the voice and facial expressions of the interviewer as well.

There are several noteworthy limitations to our study. First, while prior analyses using machine learning on audio and visual features have enrolled comparable sample sizes [19,25,48], a power analysis was not conducted given the exploratory nature of this project, and additional research with more participants is necessary to support generalizability. Second, many patients included in the project were clinically stable, experiencing mild to moderate symptoms and minimal symptom fluctuations throughout the trial, which limited our ability to assess audiovisual patterns as a function of symptom severity. It is also possible that predominant negative symptoms in our sample, such as facial blunting and lack of vocal inflection, limited our ability to detect a greater number of signs and symptoms from the BPRS, HAMD, and YMRS. Third, the effects of various medications on physiological changes in voice and facial movements in our sample remain unclear and were not taken into consideration. Further research will be needed to determine the impact of the class and dose of prescribed medications on audiovisual patterns, as well as their potential impact on behavior over the course of the interview. Furthermore, demographic variables differed among the 3 groups. Although sex differences were accounted for in our models, the potential impact of physiological differences stemming from age, race, and ethnicity (though much less likely [61,78]) warrant further exploration. Fourth, the interviewer was not blinded to diagnostic groups, which may have biased the ratings. However, the interviewer was highly trained to utilize rating scales and achieved high interrater reliability prior to study initiation. Fifth, diagnoses were clinically ascertained and extracted from the medical records. Future research should consider implementing more reliable and structured methods for diagnostic assessment, such as a structured clinical interview [79], to ensure the most accurate diagnoses. Sixth, many top features contribute to each of the best performing models, both independently and combined. Given the very large number of relevant features, we chose to emphasize and illustrate a select few in the manuscript. Corresponding clinical interpretations may, therefore, be dependent on the features highlighted and additional research will be necessary to confirm findings before clinical conclusions can be drawn. Finally, we chose to focus our analysis on acoustic components of speech rather than content as they are less dependent on cultural, socioeconomic, and educational backgrounds. Our group is, however, engaged in ongoing research aimed at the integration of speech content in the analytics framework, which we anticipate will improve our ability to detect additional psychiatric signs and symptoms.

Audiovisual data hold promise for gathering objective, scalable, noninvasive, and easily accessed, indicators of psychiatric illness. Much like an x-ray or blood test is routinely used as adjunctive data to inform clinical care, integrating audiovisual data could change the way mental health clinicians diagnose and monitor patients, enabling faster, more accurate identification of illness and enhancing a personalized approach to medicine. This would be a significant step forward for psychiatry, which is limited by its reliance on largely retrospective, self-reported data.

## Appendix

Multimedia Appendix 1 Voice features.

Multimedia Appendix 2 Facial action units.

## Abbreviations

AUROC: area under the receiver operating characteristic curve
BPRS: Brief Psychiatric Rating Scale
HAMD: Hamilton Depression Rating Scale
SANS: Scale for the Assessment of Negative Symptoms
YMRS: Young Mania Rating Scale
