# Supplementary material for: Acoustic and Facial Features From Clinical Interviews for Machine Learning–Based Psychiatric Diagnosis: Algorithm Development
Source: JMIR Ment Health. 2022 Jan 24;9(1):e24699. doi: 10.2196/24699 (PMC8822433; doi:10.2196/24699)
Supplement: Multimedia Appendix 1 [file mental_v9i1e24699_app1.docx]

Table 1. Voice Features.

| **Name (color coded)** | Description | OpenSmile Prefix |
| --- | --- | --- |
| **Voice Wavelength Features (Time Domain)** | | |
| **Jitter** | Measures the instability in frequency of the vocal cords’ vibrations. | *jitterLocal, jitterDDP* |
| **Shimmer** | Measures the instability in the amplitude of the vocal cord vibrations. | *shimmerLocal* |
| **HNR** | Harmonics to noise ratio. Quantifies the aperiodicity of the speech waveform (which can be due to additive noise) | *logHNR* |
| **Zero Crossing Rate** | Rate of sign change of voice wavelength. If the ZCR is high, the speech is unvoiced (noise), while if the ZCR is low the speech is voiced. | *pcm_zcr* |
| **Voice Spectral Features (Frequency Domain)** | | |
| **Fundamental frequency** | Main frequency component of the voice signal. It is an analog of voice pitch. Tone and intonation are both achieved through changes of fundamental frequency. | *F0final_sma* |
| **Spectral Flux** | Measures how quickly the voice power-spectrum is changing. It is an indirect measurement of the altercation of phonemes. | *pcm_fftMag_spectralFlux* |
| **Spectral Centroid** | Center of gravity of the spectrum. It is a predictor of the ‘brightness’ of a sound. The brightness quality is correlated with increased power at high frequencies. | *pcm_fftMag_spectralCentroid* |
| **Spectral Entropy** | It quantifies how tone-like a sound is, as opposed to being noise-like. Entropy is an analog of flatness. | *pcm_fftMag_spectralEntropy* |
| **Spectral Slope** | Measures how rapidly the amplitudes of successive component frequencies in the spectrum decrease as they get higher in frequency. A low spectral slope will be associated with a strident sound, whereas a high spectral slope will reflect a “fluty” sound. | *pcm_fftMag_spectralSlope* |
| **Psychoacoustic Sharpness** | Measures the high frequency content of a sound. The greater the proportion of high frequencies, the ‘sharper’ the sound. | *pcm_fftMag_psySharpness* |
| **Spectral Harmonicity** | Measures voice quality. Low SH is associated with a rough, hoarse voice. | *pcm_fftMag_spectralHarmonicity* |
| **Spectral Statistical metrics (Variance, Skewness and Kurtosis)** | Common statistics derived from the voice spectrum | *pcm_fftMag_spectralKurtosis*  *pcm_fftMag_spectralSkewness*  *pcm_fftMag_spectralVariance* |
| **Voice Spectral Envelope Features (Frequency Domain)** | | |
| **MFCCs** | Mel Frequency cepstral coefficients describes the overall shape of the spectral envelope in the mel scale of frequency. It provides a good representation of the auditory spectrum. | *pcm_fftMag_mfcc* |
| **RASTA-PLP (Relative Spectral Transform - Perceptual Linear Prediction) auditory bands** | RASTSA-PLP coefficients describes the overall shape of the spectral envelope in the Bark scale of frequency. They provide a compact representation of the auditory spectrum. | *audSpec_Rfilt* |
| **Voice Spectral Energy Features (Frequency Domain)** | | |
| **Loudness** | Subjective perception of sound pressure | *audspec_lengthL1norm, audspecRasta_lengthL1norm* |
| **Energy** | Quantifies the energy in the voice spectrum; possibly in specific frequency bands. | *pcm_RMSenergy, pcm_fftMag_fband250-650, pcm_fftMag_fband1000-4000, pcm_fftMag_spectralRollOff* |
