# Supplementary material for: Acoustic and Facial Features From Clinical Interviews for Machine Learning–Based Psychiatric Diagnosis: Algorithm Development
Source: JMIR Ment Health. 2022 Jan 24;9(1):e24699. doi: 10.2196/24699 (PMC8822433; doi:10.2196/24699)
Supplement: Multimedia Appendix 2 [file mental_v9i1e24699_app2.docx]

Table 2. Facial Action Units Description.

| Action Unit | Description |
| --- | --- |
| 1 | Inner Brow Raiser |
| 2 | Outer Brow Raiser |
| 4 | Brow Lowerer |
| 5 | Upper Lid Raiser |
| 6 | Cheek Raiser |
| 7 | Lid Tightener |
| 9 | Nose Wrinkler |
| 10 | Upper Lip Raiser |
| 12 | Lip Corner Puller |
| 14 | Dimpler |
| 15 | Lip Corner Depressor |
| 17 | Chin Raiser |
| 20 | Lip Strecher |
| 23 | Lip Tightener |
| 25 | Lips part |
| 26 | Jaw Drop |
| 28 | Lip Suck |
| 45 | Blink |
